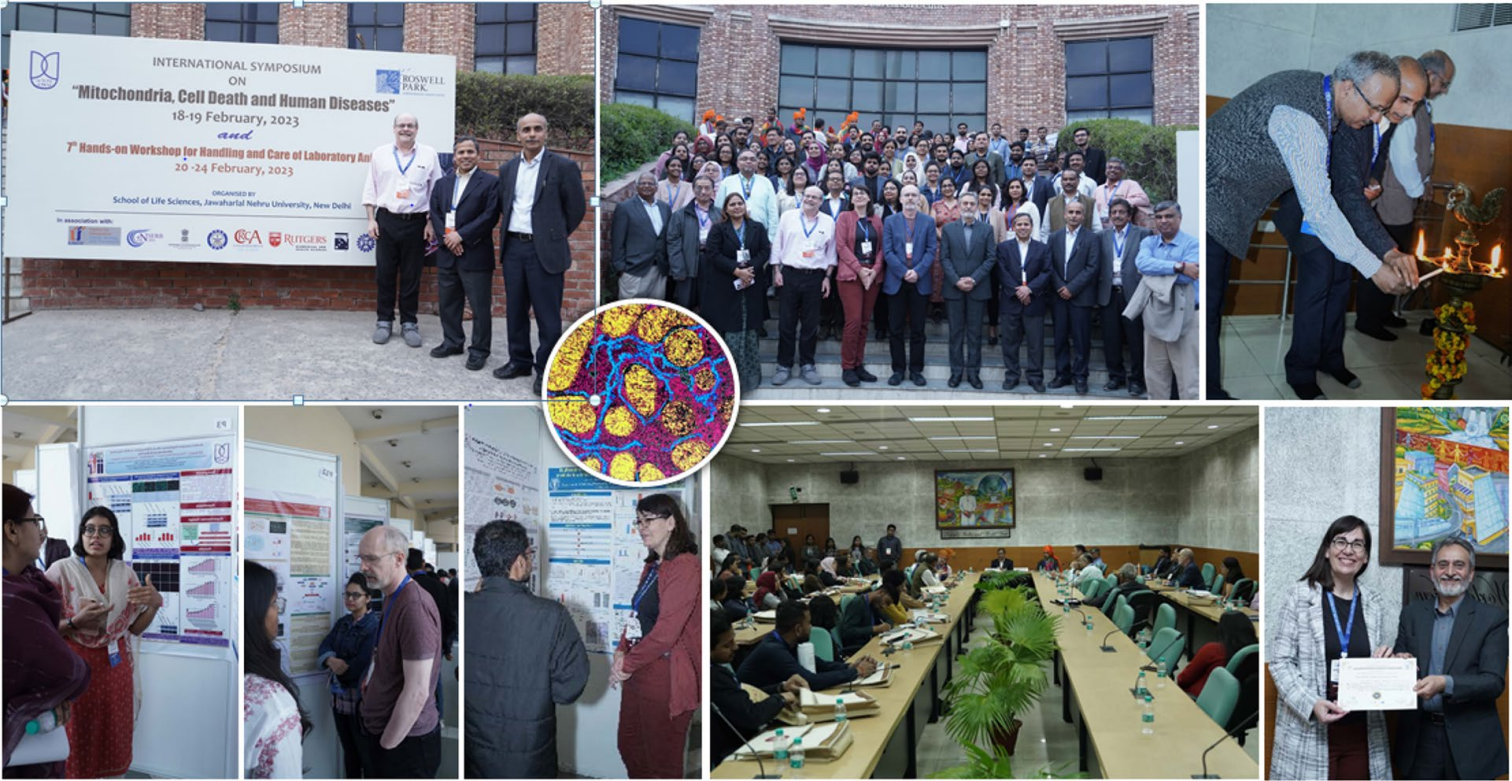# Meeting report: International Symposium on Mitochondria, Cell Death and Human Diseases: Jawaharlal Nehru University, New Delhi, India, February 18–19, 2023

**DOI:** 10.1186/s12964-023-01164-7

**Published:** 2023-06-06

**Authors:** Rana P. Singh, Raymond B. Birge, Dhyan Chandra

**Affiliations:** 1 School of Life Sciences, Jawaharlal Nehru University, New Delhi, India; 2 Rutgers University, New Jersey Medical School, Cancer Institute of New Jersey, Newark, NJ USA; 3 Pharmacology and Therapeutics, Roswell Park Comprehensive Cancer Center, Buffalo, NY USA

## Abstract

The School of Life Sciences at the Jawaharlal Nehru University in New Delhi, India held an International Symposium on Mitochondria, Cell Death and Human Diseases on February 18–19, 2023. The meeting provided a highly interactive forum for scientific discussion, cultural exchange, and collaborations between international scientists working in diverse areas of mitochondrial biology, cell death, and cancer. The two-day symposium attracted more than 180 delegates that included leading international scientists, early career researchers in India, as well as postdoctoral fellows and students. Several of the students, postdoctoral fellows, and junior faculty presented platform talks and had a chance to showcase the depth and emerging progress in biomedical research in India. The meeting will be instrumental for planning future congresses and symposium throughout India, not only to focus on mitochondrial biology, cell death and cancer but to foster continued ferment and collaborations in the biological sciences throughout India.

## Meeting highlights

The International Symposium on Mitochondria, Cell Death and Human Diseases (https://themitochondria.org/) was held in New Delhi, India, from February 18–19, 2023. The symposium was organized by Prof. Rana P Singh, School of Life Sciences, Jawaharlal Nehru University (JNU), and Cancer Research and Care Academy, New Delhi, India; Prof. Raymond B. Birge, Rutgers School of Biomedical and Health Sciences, USA; and Prof. Dhyan Chandra, Roswell Park Comprehensive Cancer Center, USA. The 2-day symposium provided an interactive platform for brainstorming current updates in the areas of mitochondria, cell death, and human diseases as well as highlighted major advances in biomedical research in many disciplines in india. The meeting was instrumental in fostering interactions between students, emerging scholars, and established leaders in mitochondrial and cell death biology. The meeting also cultivated international collaborations among established scientists in India and abroad. More than 40 senior scientists from India and USA participated in the meeting. The program included two keynote talks, five plenary talks, fifteen talks by senior scientists, and selected six talks by young faculty and five talks by young Ph.D. research scholars for the award categories. Moreover, 45 poster presentations were included on various disciplines focusing on mitochondria, cell death, and cancer in relation to human diseases. The symposium witnessed the participation of more than 180 delegates.

Major themes encompass (a) mitochondrial dynamics including fission and fusion, dysfunction, and cell death, (b) role of mitochondria in cancer, (c) mitochondria in translational research, (d) mitochondria in stem cell and regeneration, (e) role of mitochondria in aging, (f) mitochondria in metabolism, inflammation, and immunity, (g) recent advances in mitochondrial research (h) mitochondrial communication, (i) mitochondria in neurological diseases and (j) cancer therapeutics.

The symposium began on 18^th^ February with an inaugural ceremony addressed by the convenor of the symposium Prof. Rana P. Singh, School of Life Sciences and Special Center for Systems Medicine JNU, New Delhi, India. The opening ceremony had a special welcome from the co-chairs Prof. Supriya Chakraborty, Dean, School of Life Sciences, JNU, New Delhi, India; Prof. R.N.K. Bamezai, Former Professor and Dean, School of Life Sciences, JNU; Prof. Dhyan Chandra, Roswell Park Comprehensive Cancer Center, Buffalo, New York, USA; Prof. Raymond B. Birge, Rutgers School of Biomedical and Health Sciences, Newark, New Jersey, USA; Prof. S. C. Garkoti, and Rector, JNU, New Delhi, India. The special vote of thanks was delivered by Prof. Paulraj Rajamani, School of Environmental Sciences, JNU, New Delhi, India.

Scientific sessions highlighted basic and translational research focused on mitochondrial and cell death biology as well as on therapeutic vulnerabilities in cancer and other human diseases. The first session began with the keynote lecture by Prof. Marcus E. Peter, Northwestern University, Feinberg School of Medicine, Chicago, Illinois, USA. He summarized the current advances on the role of death induced by survival gene elimination (DISE)/6mer seed toxicity in cancer and neurodegenerative diseases. Prof. Raymond B. Birge, Rutgers School of Biomedical and Health Sciences, Newark, New Jersey, discussed on how phosphatidylserine exposure, stress, and cell death causes immune regulation in cancer. Prof. Dhyan Chandra, Roswell Park Comprehensive Cancer Center, Buffalo, New York, USA elaborated the importance of mitochondrial unfolded protein response in prostate cancer followed by a talk by Prof. Pankaj K. Singh, the University of Oklahoma College of Medicine, Oklahoma, USA on the role of mitochondrial metabolism and its therapeutic vulnerability in cancer. Prof. K. Thangaraj, Centre for DNA Fingerprinting and Diagnostics (CDFD) Hyderabad, Telangana, India emphasized on the dual genetic origin of neuromuscular disorders.

The second session started with a discussion on mitochondrial biology and genome. Prof. Sanjay V. Malhotra Knight Cancer Institute, Oregon Health & Science University, Portland, USA talked about targeting Guanylate Binding Protein 1 (GBP1) regulated modulation of proteasomal machinery for cancer treatment. Prof. Benu Brata Das from the Indian Association for the Cultivation of Science Kolkata, India emphasized on trapped protein-DNA covalent complexes in the mitochondria and their role in human diseases. Dr. Subhrajit Saha University of Kansas Medical Center Kansas City, Kansas, USA discussed mitochondrial biology of radiation and other anti-cancer treatment. Prof. K. Satyamoorthy, Department of Cell and Molecular Biology, School of Life Sciences, MAHE, Manipal, India, gave an overview on relevance of mitochondrial RNA:DNA hybrids during senescence in breast cancer cells. Dr. Swasti Raychaudhuri, CSIR- Centre for Cellular and Molecular Biology Hyderabad, Telangana, India, provided the importance of the inner mitochondrial membrane microenvironment on the evolution of respiratory complexes.

Several excellent talks highlighted current updates on mitochondrial bioenergetics, metabolism and communications on the second day of the meeting. Dr. Dhanasekaran Shanmugam, CSIR National Chemical Laboratory, Pune, discussed the role of mitochondrial metabolism in malaria parasites resistance followed by presentation on electron transfer activity of the mitochondrial outer membrane protein mito-NEET from Prof. Huangen Ding, Lousiana State University. Prof. Yidong Bai summarized current updates on the importance on mitochondrial dysfunction in tumorigenesis. Dr. Piya Ghose elaborated the mitochondria and endoplasmic reticulum communications in compartmentalized cell elimination. Mitochondial imaging and therapeutics aspects were discussed by Drs. Sudipta Basu and Lokendra Kumar Sharma. Mitochondrial DNA mutations in COVID-19 disease severity and mutation profile and function in neurodegenerative mitochondriopathy-LHON were introduced by Drs. Yamini Singh and P. Sundaresan, respectively. Mitochondrial targeted imaging and toxicity due to chemical threat exposure were also discussed by Drs. Samit Guha and Neera Tewari-Singh, respectively. Second keynote speaker, Dr. Jerry Chipuk, Icahn School of Medicine at Mount Sinai, New York, USA, highlighted on mitochondrial contributions to cancer: causes, consequences, and coincidence. Dr. Chipuk also discussed about the importance of shape and size of mitochondria with respect to various pathological conditions.

A final panel discussion session was led by Rana P Singh and Dhyan Chandra where panelists (Marcus Peter, Raymond Birge, Jerry Chipuk, Yidong Bai, Savita Yadav, and K Satyamoorthy) and other scientists made the meeting much more interactive and aware about the challenges and opportunities for targeting mitochondria and cell death in cancer control and management. Overall, the symposium provided a platform to share, analyze, and gain knowledge about recent research by interacting with eminent speakers and delegates as well as symposium emphasized on advances in mitochondrial biology and therapeutics in cancer and other human diseases. The meeting was concluded by award ceremony for junior faculty and young investigators. All in all, the meeting showcased the emerging growth in biomedical sciences in India, and provided a rewarding cultural and scientific exchange for all.